# Jaw exercise in head and neck cancer patients for prevention of temporomandibular disorders: a randomized controlled trial

**DOI:** 10.1007/s11764-024-01717-w

**Published:** 2024-11-22

**Authors:** Ellie Saghafi, Kalid Kadhim, Charlotte Andrén Andås, Birgitta Johansson Cahlin, Caterina Finizia, Therese Axelsson, Göran Kjeller, Lisa Tuomi

**Affiliations:** 1 Department of Oral and Maxillofacial Surgery, Region Västra Götaland, Institute of Odontology, Sahlgrenska Academy, University of Gothenburg, Medicinaregatan 12A, 413 90 Gothenburg, Sweden; 2 Department of Orofacial Pain, Institute of Odontology, Sahlgrenska Academy, University of Gothenburg, Gothenburg, Sweden; 3 Clinic of Orofacial Pain, Public Dental Service, Region Västra Götaland, Gothenburg, Sweden; 4 Department of Otorhinolaryngology, Head and Neck Surgery, Region Västra Götaland, Sahlgrenska University Hospital, Region Västra Götaland, Gothenburg, Sweden; 5 Department of Otorhinolaryngology, Head and Neck Surgery, Institute of Clinical Sciences, Sahlgrenska Academy, University of Gothenburg, Gothenburg, Sweden; 6 Institute of Neuroscience and Physiology, Speech and Language Pathology Unit, Sahlgrenska Academy, University of Gothenburg, Gothenburg, Sweden

**Keywords:** Temporomandibular disorders (TMD), Head and neck neoplasms, Radiotherapy, Symptom, Intervention, Trismus, Prevention

## Abstract

**Purpose:**

To prospectively evaluate the effect of a preventive jaw-training intervention program on the development of temporomandibular disorders (TMD) in patients treated for head and neck cancer (HNC).

**Methods:**

We randomized 58 consecutive patients with squamous cell carcinoma in the head and neck area into two groups before initiation of a curatively intended oncologic treatment: training with a jaw mobilizer once a day or a control group without active exercise. A comprehensive examination according to diagnostic criteria for temporomandibular disorders (DC/TMD) was conducted at baseline (before oncologic treatment) 6 and 12 months after completed radiation therapy (RT). The patients recorded training frequency in a diary.

**Results:**

There were significant differences in the changes of maximal incisal opening (MIO) between the intervention and control groups at 6 and 12 months compared to baseline (*p* = 0.010 and *p* = 0.012, respectively) with more deterioration in the control group. The control group had a higher prevalence of TMD diagnosis at the 6-month follow-up (*p* = 0.010) and a close to significant level at the 12-month follow-up (*p* = 0.055).

**Conclusion:**

This unique study, which evaluates the effect of a preventive jaw-training program for prevention of TMD in patients with HNC undergoing high dose RT, found that the preventive jaw-training program could prevent the deterioration of MIO and development of TMD.

**Implications for Cancer Survivors:**

This preventive exercise program could prevent the deterioration in MIO and the development of TMD in HNC patients, leading to less pain and better jaw function.

**Supplementary Information:**

The online version contains supplementary material available at 10.1007/s11764-024-01717-w.

## Background

Squamous cell carcinoma accounts for 90% of head and neck cancer (HNC) cases. HNCs are found in the oral cavity, larynx, nasal cavity, pharynx, salivary glands, or paranasal sinuses [1]. In Sweden, the incidence of cancer is increasing (> 60,000/year) with an annual incidence of > 1700 HNC patients. During the last 10 years, HNC has increased annually in both women and men by 3.3%, making it the fifth fastest increase of cancers in Sweden [2]. The main treatment modalities for HNC include radiation therapy (RT), chemotherapy, and surgical intervention, either as a single therapy or as a combined approach [3, 4]. Recent studies show that HNC survivors continue to have unmet needs in terms of supportive care services for adapting to life after treatment and managing long-term health consequences of their disease [5]. Typically, RT side effects of the head and neck area are xerostomia, mucositis, and dysphagia. Reduced skin elasticity due to subsequent fibrosis of the vessels and the subcutaneous tissues is also a known side effect [6, 7], and muscular fibrosis may result in trismus, i.e., restricted mouth opening (≤ 35 mm) [8]. Even though the mechanism behind radiation-induced trismus is not fully understood, some factors such as radiation-induced inflammation, hypoxia, endothelial injury, and fibrosis are known to contribute [9]. The cut-off for trismus have been debated, and therefore, the incidence of trismus may vary depending on what criteria used in different studies [10]. However, the definition of trismus in HNC patients widely used is a restricted mouth opening of ≤ 35 mm [11–13]. The incidence of trismus also varies significantly depending on the tumor’s size and location, with literature reporting rates ranging from 5 to 42% among patients with head and neck cancer [7, 8, 14]. Due to recent advancements in treatment technology, however, a decreased rate of trismus as low as 10%, have recently been reported [15].

Evidence suggests that trismus develops more rapidly within the first 9 months after treatment [16, 17]. Post-radiation fibrosis seems to be multifactorial. One important factor is radiation-induced damage to microvascular structures, which results in a chronic vascular and endothelial dysfunction. Subsequently, this generates leakage of pro-inflammatory factors and stimulation of fibroblasts, leading to a progressive fibrosis and sclerosis. It is widely recognized that inflammation plays a crucial role in the development and progression of trismus post-radiation treatment [14, 17, 18]. Other factors of importance seem to be both the type of radiation as well as the dose [9].

Risk factors for trismus mentioned in the literature also include the use of tobacco and alcohol, prior surgery in the head and neck area, the RT dose to the masticatory muscles, restricted mouth-opening prior to RT and volume and RT techniques [14, 19].

In addition, two individuals with the same prerequisites and treatment may develop different degrees of radiation-induced trismus. This variability in patients suggests that unknown factors play an important role in trismus development post-radiation [16, 17].

Trismus is a depressing and often painful condition that can significantly reduce the patient’s Health-Related Quality of Life (HRQoL) such as the ability to eat some foods [20]. Previous research has shown that structured jaw training improves mouth opening ability and reduces symptoms when opening the mouth and therefore improves HRQoL [21, 22]. However, only a few well-designed studies have investigated whether preventive treatment can prevent trismus or other oro-facial dysfunctions such as pain. A systematic review of the preventive outcome of incidence of trismus found no significant difference between standard care and exercise therapy adjuvant that uses a jaw-mobilizing device, and the review concludes that additional high-quality randomized controlled trials are needed [23].

Restricted mouth-opening and pain in the jaw system are conditions included in the umbrella term temporomandibular disorders (TMD). TMD also includes other musculoskeletal conditions such as pain in the temporomandibular joint (TMJ), muscles, or related structures, associated headaches, and dysfunction such as impaired jaw function, separately or combined. TMD is frequently observed in HNC patients subjected to radiotherapy, and signs and symptoms of TMD have been observed in up to 94% of patients 6 months post-radiation [24, 25].

The etiology of TMD is multifactorial and complex. Recent studies show that psychosocial factors such as personality, behavior, and environment influence the development of TMD in the general population [26]. TMD symptoms seem to be more prevalent in patients with psychosomatic symptoms. Perceived stress and previous life events may play a role in predicting TMD incidence [27, 28]. This finding could be relevant regarding the incidence of TMD in HNC patients who might be experiencing a stressful period during treatment. Other studies have shown that occlusal factors and oral parafunctions such as tooth grinding may play a role in the etiology of TMD [29–31]. Several studies have shown that the prevalence of TMD in the general population is high and is estimated to be between 5 and 15% [32, 33]. The etiology of restricted mouth-opening and pain in the masticatory system, which are common symptoms of TMD, may differ in HNC patients in comparison to the general population. It is often related to the tumor invasion or as a result of the RT to the masticatory system as previously described [19].

To our knowledge, no prior studies have evaluated the effect of preventive jaw training in regard to the development of TMD in HNC patients. Prevention is among the top priorities in both dentistry and medicine. Since TMD is frequently observed in HNC patients subjected to radiotherapy, there is a need to evaluate prevention efforts directed towards TMD symptoms in HNC patients [24, 25].

## Aim

This randomized controlled trial prospectively evaluates the effect of a preventive jaw-training intervention program on the development of TMD symptoms with respect to maximal mouth opening and muscular/TMJ pain in patients treated for HNC.

## Material and methods

### Participants

All participants were reviewed at the weekly multidisciplinary tumor board meeting at Sahlgrenska University Hospital (Gothenburg, Sweden) between 2020 and 2022. This study is part of a larger randomized study that included participants from a 2019–2022 randomized study that evaluated the effects of preventive jaw-training and swallowing exercises. Results from primary analyses are reported elsewhere [34]. Patients were eligible for inclusion if they were over 18 years old; had a recently diagnosed tumor of the oropharynx, hypopharynx, and larynx; and were treated with curative (RT) with or without chemotherapy. Patients with previous HNC diagnosis and treatment were excluded (except tonsillectomy or diagnostic sample excision) or other treatment for HNC, tracheostomy. Patients with neurological, or neuromuscular disease, or cognitive impairments (e.g., language difficulties, reading difficulties, or dementia) that made it impossible for patients to answer questionnaires were also excluded. In addition, patients were excluded if they had a history of trismus or trismus at diagnosis (MIO ≤ 35 mm), substandard dental status (i.e., patient could not use jaw trainer), or impaired general condition or other illness. Patients who met the criteria for inclusion were asked to participate in the study. Those who agreed to participate were randomized to either the preventive jaw training group or the control group.

### Design

This single-blinded randomized controlled trial consisted of a baseline assessment of pain and function of the jaw system according to a standardized diagnostic criterion for temporomandibular disorders with a follow-up assessment 6 and 12 months post treatment. The computerized randomization was performed through optimal allocation according to Pocock’s sequential randomization method regarding tumor site, tumor stage, HPV status, age, sex, comorbidity (Adult Comorbidity Evaluation-27), swallowing ability, and MIO [35]. Patients were randomized into either an intervention group receiving preventive jaw-training or a control group receiving standard care alone according to clinical practice. According to an 80% power calculation (Mann–Whitney *U* test, alpha = 0.05), the sample size was calculated to 24 patients in each group (*n* = 48 in total), assuming a clinically relevant difference in MIO of 5 mm (SD = 5.8). We aimed to include 58 patients taking possible dropouts into account. The power calculation was based on previous research results.

### Oncological treatment

All patients received treatment in accordance with the regional and national cancer treatment program [1]. Curative (RT) was given as volumetric modulated radiation therapy (VMAT) in a moderately accelerated fractionation schedule to a total dose of 68 Gy in 2 Gy fractions once or twice daily for six treatments per week [15]. A majority of the patients also received chemotherapy as concomitant therapy [36]. Concomitant chemotherapy generally consisted of six cycles of cisplatin. Eight patients received no chemotherapy. No statistically significant differences were found between the groups regarding oncologic treatment received.

### Intervention

The intervention consisted of active and passive jaw training using a mechanical jaw exercise device, JawTrainer ™. Passive stretching was done with the jaw trainer for 30 s three times with 20–25 s rest between use. Active training requires biting against the resistance provided by the Jaw Trainer for a couple of seconds. This training was repeated five times. The training was scheduled once a day from diagnosis, during RT, and up to 1 month post treatment. The participants were recommended to continue the training program for at least three times/week during the whole follow-up period. All patients in both intervention and control group had weekly contact with study personnel during and up to approximately 1 month after completion of RT. The control group was mainly asked about potential symptoms and ability to eat and drink, while the intervention group also was asked about the jaw exercises. All the participants in the intervention group recorded their impressions in a diary regarding the training and their compliance with training during the RT. Additionally, a concluding question was asked at each follow-up: On average, how many times a day do you practice? All participants in both intervention and control group received information about possible side effects of the RT, including the possibility of reduced mouth-opening ability and pain in the jaw system. The intervention also included a swallowing exercise, which has been described elsewhere [34]. Following oncologic treatment, all patients in both intervention and control group who reached a mouth opening of 35 mm or below received jaw training according to current best evidence about trismus intervention with a recommendation of training with the Jaw Trainer™ three times/day and no longer following the intervention or control group instructions [37, 38].

### Examination

At baseline, all patients received a full TMD examination based on Diagnostic Criteria for Temporomandibular Disorders (DC/TMD) [39] to assess symptoms indicative of TMD. The same type of examination was performed 6 and 12 months after completion of oncologic treatment by one of two examiners (authors ES or KK). The study was single-blinded as the examiner did not know if the patients were in the intervention group or the control group. The two examiners were calibrated regarding intra- and inter-reliability according to DC/TMD Training and Calibration recommendations from the International RDC-TMD Consortium [39].

Axis I, a comprehensive DC/TMD clinical examination protocol, involves ten examination items: location of subjective pain, incisal relationship, opening movements in millimeters, lateral and protrusive movements in millimeters, jaw opening pattern, TMJ noise during opening/closing/lateral/protrusive movements, and joint locking. Measurements (in mm) of maximal interincisal opening (MIO), which is the maximal distance between the edges of the incisors of the mandible and the maxilla, were made with a ruler with the patient sitting in an upright position. The patients were instructed to maintain a neutral head posture throughout the procedure and to open their mouth as wide as they could. The MIO measurements were recorded at the maximum opening without assistance. Muscle palpation sites include the masseter, temporalis, and supplemental muscles such as the lateral pterygoid area and the posterior and submandibular muscles. The calibrated palpation of muscle and TMJ was used to assess pain upon palpation. All registrations were done separately for the left and the right side of the face [39]. A short and focused Symptom Questionnaire (DC/TMD SQ) was used to assess pain characteristics as well as history of jaw noise, jaw locking, and headache. The DC/TMD SQ is a patient-reported questionnaire that provides the necessary history for the Axis I diagnostic criteria [39].

Five common clinical TMD-related diagnoses according to DC/TMD were evaluated in this study: myalgia, arthralgia, headache attributed to TMD, disc displacement with or without reduction, and degenerative joint disease. In addition, the Graded Chronic Pain Scale (GCPS) was used, which is part of Axis II instruments in the DC/TMD, as these are reliable self-reported instruments used for evaluation of psychosocial status. GCPS is mainly used for assessing pain intensity. The Oncology Section EDGE Task Force on Head and Neck Cancer Outcomes also recommends GCPS for clinical use as it found that patient-reported outcome measures from individuals with HNC-related temporomandibular dysfunction to be effective [40].

GCPS is a short, reliable, and validated instrument that assesses pain intensity and pain-related disability [41]. The GCPS includes two subscales. One subscale, characteristic pain intensity (CPI), reliably measures pain intensity and is comprised of the mean value of three measures—the intensity of current, average, and worst clinical pain over the past month with scores ranging from 0 to 10 using a numeric rating scale (NRS) where 0 is no pain and 10 is the worst thinkable pain. The other subscale, the pain-disability rating, is based on the number of days that pain interferes with activity and on the extent of interference with social, work, or usual daily activities [42].

### Statistics

All statistical analyses were performed using SPSS version 28.0.1.0 (142). Two-sided tests were used for all analyses and* p* < 0.05 was considered to be statistically significant. For descriptive purposes, the mean, standard deviation, median, and range are presented. For categorical variables, numbers and percentages are presented. For comparison between control and intervention group, Fisher’s exact test was used for dichotomous variables, and Mann–Whitney *U* test was used for continuous variables. In the intention-to-treat (ITT) analysis, multiple imputation was applied to the dataset using SPSS for the missing values for the primary endpoints. The number of imputations were five and independent samples *t*-test were used to determine a pooled *p*-value from the imputed datasets together with the original dataset. The method for imputation was linear regression model based on the baseline variables as the randomization and the tested endpoint at baseline. The primary measure endpoint was the MIO according to the DC/TMD protocol, and the primary patient-reported endpoint was changes in the pain scale (NRS) and in the face/jaw or mouth area (CPI). Secondary measures were the five DC/TMD diagnoses: myalgia, arthralgia, headache attributed to TMD, disc displacement with reduction, disc displacement without reduction, and degenerative joint disease, which are based on both clinical measures and patient-reported outcomes. For the sensitivity analysis, a per protocol analysis included participants in the intervention group who exercised at least 50% of the recommended exercise dose and participants without trismus as the participants who developed trismus were offered a different exercise protocol. The per protocol analysis was performed for 13 participants in the intervention group and 19 in the control group.

### Ethics

This study was approved by the Swedish Ethical Review Authority (1151–18/2019- 00752 and 2020–04581). The study was conducted according to the Declaration of Helsinki, and all participants gave their written informed consent to participate.

## Results

### Participants

**Fig. 1:**
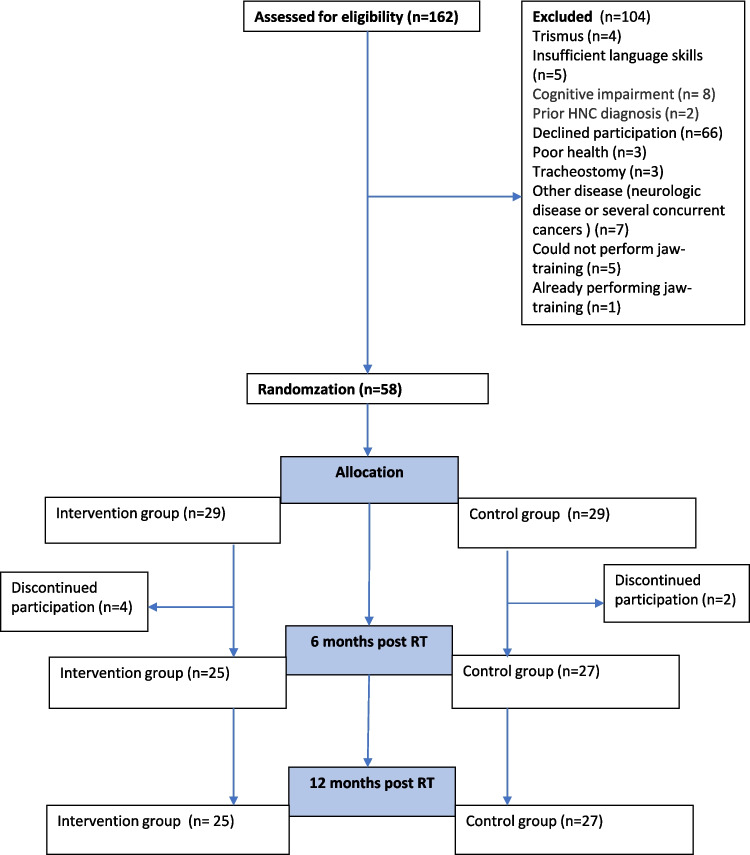
Flow chart showing participants that were assessed for eligibility

From January 2020 through November 2022, a total of 162 patients were assessed for eligibility, and 104 of these patients were deemed not eligible and therefore excluded (Fig. 1). The 58 remaining patients agreed to participate in the study and were randomized into a preventive exercise group (*n* = 29) and a control group (*n* = 29). All 58 participants who were randomized were included in the baseline examinations. At the 12-month follow-up, 52 participants were still enrolled.

Table 1 lists the patient characteristics at baseline. No statistically significant differences were found between the groups for sex, age, comorbidities, tumor localization, classification or stage, radiotherapy regimen, or the measured endpoints. All patients were residents of the same region and treated at the same center. Drop-out analyses were performed. The patients who were randomized but did not complete their participation were compared with the patients included in the intervention and control groups, respectively. The analyses did not reveal any statistically significant differences in any of the variables reported in Table 1.

**Table 1:** Baseline variables

Baseline variable	Intervention *n* = 29	Control *n* = 29	*p*-value
	Mean (SD) Median (Min; Max)	Mean (SD) Median (Min; Max)	
Age (years)	68.5 (9.0) 68 (46; 83)	65.8 (10.6) 67 (42; 85)	> 0.30
	(%) †	(%) †	
Sex			
Male	20 (69.0)	19 (65.5)	> 0.30
Female	9 (31.0)	10 (34.5)	
Education level			
Elementary	8 (29.6)	5 (21.7)	> 0.30
High school	9 (33.3)	8 (34.8)	
College/university	9 (33.3)	9 (39.1)	
Missing value	1 (3.7)	1 (4.3)	
Tumor location			
Tonsil	14 (48.3)	20 (69.0)	0.13
Base of tongue	13 (44.8)	6 (20.7)	
Hypopharynx	2 (6.8)	3 (10.3)	
Tumor stage			
I	15 (51.7)	15 (51.7)	> 0.30
II	3 (10.3)	1 (3.4)	
III	8 (27.6)	6 (20.7)	
IV	3 (10.3)	7 (24.1)	
Radiotherapy	29 (100.0)	29 (100.0)	
Comorbidity according to ACE-27*			
None, mild	22 (75.9)	23 (79.3)	> 0.30
Moderate, severe	7 (24.1)	6 (20.7)	
Chemotherapy (concomitant)	26 (89.7)	24 (82.8)	> 0.30
Chemotherapy cycles			
0	3 (10.3)	6 (20.7)	0.19
3	0 (0.0)	5 (17.2)	
4	6 (20.7)	3 (10.3)	
5	9 (31.0)	8 (27.6)	
6	11 (37.9)	7 (24.1)	

^†^Percentages rounded (i.e., does not always sum to 100)

*ACE-27, Adult Comorbidity Index

### MIO and trismus

The primary analysis revealed that there were statistically significant differences between the intervention and control group regarding change in MIO from baseline to 6 months (*p* = 0.010) and to 12 months (*p* = 0.012) (Table 2). The control group presented with a greater deterioration of MIO, which was reduced by 8.2 mm and 6.0 mm at the 6- and 12-month follow-ups, respectively, than the intervention group, which had a reduction of only 2.8 mm and 1.2 mm at the 6- and 12-month follow-ups, respectively. Sensitivity analyses of complete case (CC) analysis and per protocol (PP) revealed similar results (Table 2 and Supplementary Table 1).

**Table 2:** Intention-to-treat and complete case

Intervention group (*n* = 29)						Control group (*n* = 29)					Comparison of intervention and control group			
											ITT with multiple imputation		Complete cases: without imputation	
	Baseline	6M	1Y	Change baseline to 6M	Change baseline to 1Y	Baseline	6M	1Y	Change baseline to 6M	Change baseline to 1Y	Difference baseline to 6M	Difference baseline to 1Y	Difference baseline to 6M	Difference baseline to 1Y
	Mean (SD) Median (Min; Max)					Mean (SD) Median (Min; Max)					Two-sided *p*-value			
MIO	47.7 (6.4) 47 (36; 60) *n* = 29	45.1 (6.9) 45 (35; 60) *n* = 25	46.7 (7.0) 45 (35; 60) *n* = 25	− 2.8 (5.9) − 1 (− 18; 6) *n* = 25	− 1.2 (6.5) 0 (− 18; 19) *n* = 25	48.4 (6.1) 50 (40; 61) *n* = 29	40.6 (9.9) 40 (11; 60) *n* = 27	42.8 (8.0) 40 (25; 60) *n* = 27	− 8.2 (7.2) − 8 (− 38; 0) *n* = 27	− 6.0 (5.4) − 6 (− 24; 0) *n* = 27	0.010	0.012	< 0.001	< 0.001
NRS	1.3 (2.1) 0 (0; 7) *n* = 28	0.9 (1.7) 0 (0; 6) *n* = 24	0.6 (1.7) 0 (0; 6) *n* = 25	− 0.2 (2.4) 0 (− 7; 6) *n* = 23	− 0.5 (2.9) 0 (− 7;6) *n* = 24	1.4 (1.5) 1 (0; 5) *n* = 29	3.1 (2.9) 3 (0; 8) *n* = 27	1.8 (2.0) 1 (0; 5) *n* = 27	1.8 (3.1) 1 (− 4; 8) *n* = 27	0.5 (2.3) 0 (− 3; 5) *n* = 27	0.015	0.13	0.012	0.22
CPI	2.3 (3.5) 0 (0; 14) *n* = 28	0.9 (1.9) 0 (0; 6.6) *n* = 23	0.5 (1.4) 0 (0; 5.5) *n* = 25	− 1.2 (3.1) 0 (− 8.2; 6.6) *n* = 22	− 1.4 (3.5) 0 (− 8.2; 5.5) *n* = 24	2.1 (2.5) 1 (0; 8) *n* = 29	2.6 (2.3) 2.5 (0; 6.3) *n* = 25	1.4 (1.6) 1.1 (0; 4.3) *n* = 27	0.3 (3.4) 0 (− 8; 6.2) *n* = 25	− 0.7 (3.0) 0 (− 8; 4.3) *n* = 27	0.061	0.28	0.034	0.46

*CPI*, characteristic pain intensity, higher CPI meaning higher average of pain intensity over the past month; *MIO*, maximal interincisal opening; *NRS*, numeric rating scale; higher NRS meaning higher pain intensity

The incidence of trismus at the 6-month follow-up was 8% (*n* = 2) in the intervention group and 29.6% (*n* = 8) in the control group. Another 4.4% (*n* = 1) in the intervention group compared to 5.3% (*n* = 1) in the control group developed trismus between the 6- and 12-month follow-ups. The occurrence of trismus at the 12-month follow-up was 12% (*n* = 3) in the intervention group and 33.3% (*n* = 9) in the control group, resulting in a total occurrence of 23% (*n* = 12). The incidence of trismus was not statistically significant between the groups at the 6-month follow-up (*p* = 0.073) or 12-month follow-up (*p* = 0.42). The incidence of trismus in total from baseline was also not statistically significant between the groups (*p* = 0.10).

### NRS and CPI

For NRS change from baseline, the control group reported a greater increase in pain intensity between baseline and the 6-month follow-up compared with the intervention group (*p* = 0.015). However, this difference between the groups did not remain at the 12-month follow-up *(p* = 0.13). The sensitivity analysis showed similar results (Table 2).

For CPI change, which includes the pain intensity in the last month, from baseline, the results were close but not significant between the groups at the 6- and 12-month follow-ups in the ITT (*p* = 0.061). In the CC analysis, the difference between the groups was statistically significant (*p* = 0.034) with higher pain intensity as measured by the CPI in the control group. No significant difference of the CPI compared to baseline was seen between the groups at the 12-month follow-ups in the ITT or the CC (Table 2).

### DC/TMD diagnoses

There was a statistically significant higher prevalence of any DC/TMD diagnosis in the control group at the 6-month follow-up (*p* = 0.010) and close to a significant level at the 12-month follow-up (*p* = 0.055) (Table 3).

**Table 3:** DC/TMD diagnosis at the examination occasions in both control and intervention group

	Intervention group			Control group			Comparison of intervention and control group Complete cases: without imputation	
	PRE *n* = 29	6M *n* = 25	1Y *n* = 25	PRE *n* = 29	6M *n* = 27	1Y *n* = 27	6M	1Y
	*n* (%)			*n* (%)			p value	
No diagnosis	17 (59)	15 (60)	18 (72)	18 (62.1)	6 (22)	12 (44)	0.010	0.055
Myalgia	3 (10)	3 (12)	2 (8)	3 (10.3)	19 (70)	12 (44)	< 0.001	0.0044
Arthralgia	2 (7)	2 (8)	1 (4)	1 (3.4)	5 (19)	4 (15)	> 0.30	> 0.30
Disk displacement with reduction	6 (21)	4 (16)	3 (12)	5 (17.2)	4 (15)	3 (11)	> 0.30	> 0.30
Disk displacement without reduction	1 (3)	1 (4)	0 (0)	1 (3.4)	1 (4)	1 (4)	> 0.30	> 0.30
Headache attributed to TMD	0 (0)	0 (0)	0 (0)	0 (0.0)	1 (4)	1 (4)	> 0.30	> 0.30
Degenerative joint disease	4 (14)	2 (8)	2 (8)	3 (10.3)	3 (11)	3 (11)	> 0.30	> 0.30

*M* months, *Y* year

The most common diagnosis of pain in the jaw system was myalgia. There was a significantly higher prevalence of myalgia in the control group both at the 6-month (*p* < 0.001) and the 12-month follow-up (*p* = 0.004). For other diagnoses, no significant differences between the groups were demonstrated at either the 6-month or 12-month follow-ups.

### Compliance to the preventive exercises in the intervention group

In a daily diary, all participants in the intervention group assessed their compliance and experience with the jaw training during the RT. The participants in the intervention group performed the jaw exercise to 69% of the recommended exercise dose. Table 4 shows the proportion of recommended dose as presented in the diary during RT. No adverse symptoms or events were reported related to the jaw training.

**Table 4:** Compliance to the preventive exercises in the intervention group from diagnosis to 1 month post-radiation therapy presented as proportion of recommended exercise dose in percent

Variable	Mean (SD) Median (Min; Max)
Performed amount of recommended passive jaw exercise	60.3 (34.7) 64 (0–100) *N* = 23
Performed amount of recommended active jaw exercise	69.0 (26.9) 64 (20–100) *N* = 23
Total performed amount of recommended jaw exercise	69.2 (27.0) 64 (20–100) *N* = 23

At the end of each follow-up, the following question was asked: On average, how many times a day do you practice? For the 6-month follow-up, 23 of 25 (92%) and at 12-month follow-up 20 of 25 (80%) participants in the intervention group reported that they trained the recommended dose (i.e., 1 time per day).

## Discussion

This prospective single blinded randomized study intended to evaluate the effect of a preventive jaw training intervention program on the development of TMD symptoms and trismus in patients treated for HNC in a preventive intervention group compared with a control group. This study showed that the preventive training with the jaw trainer in HNC patients undergoing RT statistically significantly affected the degree of mouth opening at the 6-month and 12-month follow-ups post RT. The participants in the control group, who did not use the jaw trainer, showed a greater deterioration of MIO and close to a significant level for the incidence of trismus with a higher prevalence in the control group. This finding is in line with recently published results from a meta-analysis of randomized controlled trials, which found that, compared with the control group, the exercise group exhibited superior mouth opening improvement over the short (5–10 weeks) and long term (3 months) [23]. The meta-analysis revealed that the exercise did not have a significant benefit in reducing the incidence of trismus, a finding that is in line with our study, i.e., the incidence of trismus was similar in both groups throughout the study. However, the prevalence, even though not statistically significantly different between the groups, revealed a percentage in the control group more consistent with the incidence of trismus in previous research, and the incidence in the intervention group was lower. It is possible that a larger study sample would have revealed a statistically significant difference.

Although there was a notable statistically significant difference in the changes of MIO between the groups in this study, the participants in both groups maintained a fairly good MIO over time. Impact of trismus or decreased MIO in terms of HRQoL was not part of this study’s scope. Trismus with a reduced MIO (≤ 35 mm) has previously been proven to be clinically relevant for HRQoL [8].

Previous studies have shown that high RT dosage (> 60 Gy) to the jaw muscles can be seen as a risk factor for a decrease in MIO [43]. The incidence of trismus in this study was a total of 23% during the first 12 months following radiotherapy. This is somewhat lower than previous reports, which found a prevalence of 30–40% [7]. The lower incidence of trismus could be due to more modern RT techniques. IMRT and VMAT treatment techniques reduce normal tissue dosage, which might result in a lower incidence of radiation-induced trismus [11, 34, 44]. Even though late subcutaneous fibrosis is less common with IMRT technique [45], it is still a relatively common cause of trismus. Therefore, a long-term follow-up at 3 and 5 years post treatment, respectively, is planned to analyze the incidence of trismus and the effect of jaw exercise in a longer perspective.

The prevalence of pain in the jaw system with the diagnosis of myalgia was significantly higher in the control group at both follow-ups. This finding indicates that jaw-mobilization exercise during radiotherapy for HNC not only prevents deterioration of jaw opening function but also reduces the risk of TMD and pain to some extent. This finding is important for both the patients with HNC and the clinicians working with this group as there is a possibility to reduce pain in the jaw system post RT and avoid malnutrition, weight loss, and chronic pain. Both groups exhibited a peak in the frequency of myalgia at the 6-month follow-up and an improvement at the 12-month follow-up, a finding that agrees with other studies where pain in the jaw muscles was evaluated [24, 25]. These studies also revealed a high prevalence of both objective and subjective signs of TMD such as pain or stiffness of the jaw and masticatory muscles as well as reduced ability of HNC patients to open their mouth and/or facial pain up to 1 year post RT.

At baseline, the prevalence of TMD diagnosis in this cohort was 41% in the intervention group and 38% in the control group, which is higher than in the general population. The TMD prevalence in the general population is typically between 5 and 15% [33]. Therefore, it could be assumed that some of the HNC patients had TMD-related pain and dysfunction problems before the malignant diagnosis [46]; however, occurrence beyond the expected within the normal population can probably be attributed to the HNC diagnosis. In addition, the patients in this study were assessed shortly after being diagnosed with a malignant disease, which could be one reason for some of the initially high frequency of a TMD diagnosis as the worries and insecurity could increase the tension of the muscles in the jaw system [27].

The prevalence of TMD depends on many factors such as diagnostic criteria. In this study, we have used a well-known and validated diagnostic criteria for temporomandibular disorders. When using patient-reported signs and symptoms of TMD rather than a valid diagnostic system such as DC/TMD, there is a higher prevalence of TMD (68%) [24]. To our knowledge, no prior studies have used a standardized diagnostic system such as DC/TMD to provide new insights into the field of TMD research in this patient group.

A recent study concluded that jaw exercises are an effective treatment for reducing pain intensity with masticatory myofascial pain in patients with TMD [47]. This study shows a significantly lower prevalence of pain in the intervention group that performed the jaw exercises. Jaw exercises are also cost-effective compared with treatment with a stabilization appliance in patients with TMD [47–49]. In this study, we used the JawTrainer ™, an instrument similar to Engström’s jaw mobilizing device, which has been shown to be equally effective in treating trismus in patients treated for HNC as Therabite® and significantly improves the mouth opening capacity of patients [21].

It seems that the low-intensity jaw-training program of 1 time/day was manageable for the patients in this cohort even though the treatment for HNC is demanding. Not all the participants reported their compliance and experiences in their diaries 1 month post RT, but most participants reported high compliance in the questionnaires at the follow-ups. According to the participants diaries, the compliance to the recommended exercise was as high as 69% during RT. Other studies have shown that the participants exercise between 24 [50] and 54% of the recommended dose where the recommended dose was 3 times/day during and up to 6 months post RT [11]. A meta-analysis of randomized controlled trials of exercise for prevention of trismus in patients with HNC concluded that risk of trismus in patients who received telephone support for their exercise regime was significantly lower than those who received Treatment-As-Usual or jaw exercise without telephone support [51]. In this study, we modified the training program so that it would be easier to maintain during their RT and the post treatment with one training session a day. We also had weekly follow-ups with the patients during their RT. These modifications, i.e., providing regular support and monitoring, could have resulted in better adherence to the exercise program.

## Strengths and limitations

This single blinded randomized controlled trial focused on two comparable groups with clear inclusion and exclusion criteria. A standardized and well-known method for diagnostics of temporomandibular disorders was used, which ensures a high specificity and sensitivity. The diagnosis is based on both patient-reported outcomes and clinical findings, which strengthens the diagnosis. The two researchers who performed the examinations were blinded to the randomization and well calibrated according to the criteria for DC/TMD.

A limitation of this study may be that all patients who reached a MIO below ≤ 35 mm were given the standardized training program, i.e., training with a jaw trainer three times/day. This schedule could have affected the results; however, ethical concerns required that we provide adequate treatment to patients with trismus. The sensitivity analysis, where the patients with trismus were excluded, revealed similar results as the ITT.

The dropouts in this study were relatively small compared to similar studies. Drop-out analysis did not reveal any statistically significant differences in any of the variables reported between the dropouts and the other participants.

The number of patients randomized to each group can be seen as relatively low, which could affect the statistical outcomes, especially since the incidence of trismus was generally low in this study.

Assessments related to swallowing and chewing functions have not been addressed in this study and could be seen as a limitation. Prior trauma, ankylosis or inflammatory diseases affecting the TMJ where not part of the inclusion criteria and could also be seen as a limitation.

## Conclusions

This study concludes that the preventive jaw-training program could help prevent the deterioration of MIO and development of TMD up to 6 and 12 months after RT. According to Djikstra et al. (i.e., MIO ≤ 35 mm) [8], the preventive exercise program did not result in differences regarding the incidence of trismus. Additionally, the incidence was somewhat lower than reported in prior studies.

## Clinical implications

This study indicates that patients with HNC undergoing RT should be informed of possible changes in MIO during RT and up to 1 year after completed RT. Furthermore, patients should be instructed to perform exercises to maintain jaw opening function during oncologic treatment. The training program could be offered to prevent temporomandibular disorders.

## Supplementary Information

Below is the link to the electronic supplementary material.

Supplementary file1 (DOCX 14 KB)

## Data Availability

No dataset generated and analyzed in this study are publicly available due to ethical reasons.

## References

[1] RCC. Regionalt Cancercentrum Väst. Nationellt vårdprogram Huvud- och Halscancer. Göteborg, 2015. http://www.cancercentrum.se/globalassets/cancerdiagnoser/huvudoch-hals/vardprogram/natvp_huvud-hals_v1.0_150825_final.pdf

[2] Nationell kvalitetsregisterrapport huvud- och halscancer The Swedish Head and Neck Cancer Register. 2022: Available from: arsrapport_2022_swehncr.pdf (cancercentrum.se)

[3] Tao Y, Daly-Schveitzer N, Lusinchi A, Bourhis J. Advances in radiotherapy of head and neck cancers. Curr Opin Oncol. 2010;22(3):194–9. 20401975 10.1097/cco.0b013e3283388906

[4] Gibson MK, Forastiere AA. Multidisciplinary approaches in the management of advanced head and neck tumors: state of the art. Curr Opin Oncol. 2004;16(3):220–4. 15069316 10.1097/00001622-200405000-00005

[5] Crowder SL, Najam N, Sarma KP, Fiese BH, Arthur AE. Quality of life, coping strategies, and supportive care needs in head and neck cancer survivors: a qualitative study. Support Care Cancer. 2021;29(8):4349–56. 33415365 10.1007/s00520-020-05981-1 PMC9186018

[6] Chua DT, Tian Y, Wei WI. Late oral complications following radiotherapy for head and neck cancers. Expert Rev Anticancer Ther. 2007;7(9):1215–24. 17892422 10.1586/14737140.7.9.1215

[7] Pauli N, Johnson J, Finizia C, Andrell P. The incidence of trismus and long-term impact on health-related quality of life in patients with head and neck cancer. Acta Oncol. 2013;52(6):1137–45. 23193958 10.3109/0284186X.2012.744466

[8] Dijkstra P, Huisman P, Roodenburg J. Criteria for trismus in head and neck oncology. Int J Oral Maxillofac Surg. 2006;35(4):337–42. 16280237 10.1016/j.ijom.2005.08.001

[9] Travis EL. Organizational response of normal tissues to irradiation. Semin Radiat Oncol: Elsevier; 2001. p. 184–96. 10.1053/srao.2001.25243 11447575

[10] Somay E, Kucuk A, Yilmaz B, Pehlivan B, Selek U, Topkan E. Definitions of radiation-induced trismus in head and neck cancer: current concepts and controversies. Exon Publications. 2022:23–40. 37756424

[11] Loorents V, Rosell J, Karlsson C, Lidbäck M, Hultman K, Börjeson S. Prophylactic training for the prevention of radiotherapy-induced trismus–a randomised study. Acta Oncol. 2014;53(4):530–8. 24635110 10.3109/0284186X.2014.892211

[12] Weber C, Dommerich S, Pau HW, Kramp B. Limited mouth opening after primary therapy of head and neck cancer. Oral Maxillofac Surg. 2010;14:169–73. 20358238 10.1007/s10006-010-0220-2

[13] Kamstra J, Dijkstra P, Van Leeuwen M, Roodenburg J, Langendijk J. Mouth opening in patients irradiated for head and neck cancer: a prospective repeated measures study. Oral Oncol. 2015;51(5):548–55. 25703798 10.1016/j.oraloncology.2015.01.016

[14] Raj R, Thankappan K, Janakiram C, Iyer S, Mathew A. Etiopathogenesis of trismus in patients with head and neck cancer: an exploratory literature review. Craniomaxillofac Trauma Reconstr. 2020;13(3):219–25. 33456691 10.1177/1943387520917518 PMC7797966

[15] Pettersson N, Andersson E, Pauli N, Tuomi L, Finizia C, Olsson CE. Decreased rates of radiation-induced trismus and lowered mastication structure doses in patients treated for head and neck cancer during the last two decades. Clin Oncol. 2024;36(10):e388–97. 10.1016/j.clon.2024.07.003 39095285

[16] Stubblefield MD. Radiation fibrosis syndrome: neuromuscular and musculoskeletal complications in cancer survivors. PM&R. 2011;3(11):1041–54. 22108231 10.1016/j.pmrj.2011.08.535

[17] Kent ML, Brennan MT, Noll JL, Fox PC, Burri SH, Hunter JC, et al. Radiation-induced trismus in head and neck cancer patients. Support Care Cancer. 2008;16(3):305–9. 17965892 10.1007/s00520-007-0345-5

[18] Somay E, Yilmaz B, Topkan E, Kucuk A, Pehlivan B, Selek U. Initial neutrophil-to-lymphocyte ratio predicts radiation-induced trismus in parotid gland cancer. Oral Dis. 2023;29(7):2772–9. 36349491 10.1111/odi.14429

[19] Hague C, Beasley W, Garcez K, Lee LW, McPartlin A, McWilliam A, et al. Prospective evaluation of relationships between radiotherapy dose to masticatory apparatus and trismus. Acta Oncol. 2018;57(8):1038–42. 29630433 10.1080/0284186X.2018.1459047

[20] Aghajanzadeh S, Karlsson T, Tuomi L, Engström M, Finizia C. Trismus, health-related quality of life, and trismus-related symptoms up to 5 years post-radiotherapy for head and neck cancer treated between 2007 and 2012. Support Care Cancer. 2023;31(3):166. 36781552 10.1007/s00520-023-07605-w PMC9925520

[21] Pauli N, Fagerberg-Mohlin B, Andréll P, Finizia C. Exercise intervention for the treatment of trismus in head and neck cancer. Acta Oncol. 2014;53(4):502–9. 24175896 10.3109/0284186X.2013.837583

[22] Aghajanzadeh S, Karlsson T, Tuomi L, Finizia C. The effect of jaw exercises on anxiety and depression in patients with head and neck cancer receiving radiotherapy: Prospective 2-year follow-up study. Head Neck. 2020;42(2):330–5. 31755605 10.1002/hed.26012

[23] Shao C-H, Chiang C-C, Huang T-W. Exercise therapy for cancer treatment-induced trismus in patients with head and neck cancer: a systematic review and meta-analysis of randomized controlled trials. Radiother Oncol. 2020;151:249–55. 32890607 10.1016/j.radonc.2020.08.024

[24] Pauli N, Mejersjö C, Fagerberg-Mohlin B, Finizia C. Temporomandibular disorder in head and neck cancer patients undergoing radiotherapy: clinical findings and patient-reported symptoms. Head Neck. 2019;41(10):3570–6. 31313400 10.1002/hed.25878

[25] Saghafi E, Tuomi L, Kjeller G. The prevalence and symptoms of temporomandibular disorders in head and neck cancer patients. Acta Odontol Scand. 2022;80(4):252–7. 10.1080/00016357.2021.1991470. 34651551 10.1080/00016357.2021.1991470

[26] Fillingim RB, Ohrbach R, Greenspan JD, Knott C, Diatchenko L, Dubner R, et al. Psychological factors associated with development of TMD: the OPPERA prospective cohort study. J Pain. 2013;14(12):T75–90. 24275225 10.1016/j.jpain.2013.06.009 PMC3855656

[27] Fillingim RB, Ohrbach R, Greenspan JD, Knott C, Dubner R, Bair E, et al. Potential psychosocial risk factors for chronic TMD: descriptive data and empirically identified domains from the OPPERA case-control study. J Pain. 2011;12(11):T46–60. 22074752 10.1016/j.jpain.2011.08.007 PMC3233685

[28] Suvinen TI, Nyström M, Evälahti M, Kleemola-Kujala E, Waltimo A, Könönen M. An 8-year follow-up study of temporomandibular disorder and psychosomatic symptoms from adolescence to young adulthood. J Orofac Pain. 2004;18(2). 15250432

[29] Magnusson T, Egermark I, Carlsson GE. A prospective investigation over two decades on signs and symptoms of temporomandibular disorders and associated variables. A final summary. Acta Odontol Scand. 2005;63(2):99–109. 16134549 10.1080/00016350510019739

[30] Glaros AC, Tabacchi KN, Glass EG. Effect of parafunctional clenching on TMD pain. J Orofac Pain. 1998;12(2). 9656892

[31] Marklund S, Wänman A. Risk factors associated with incidence and persistence of signs and symptoms of temporomandibular disorders. Acta Odontol Scand. 2010;68(5):289–99. 20528485 10.3109/00016357.2010.494621

[32] Nilsson I-M, List T, Drangsholt M. Incidence and temporal patterns of temporomandibular disorder pain among Swedish adolescents. J Orofac Pain. 2007;21(2):127–32. 17547124

[33] LeResche L. Epidemiology of orofacial pain. Orofacial pain. From basic science to clinical management. The transfer of knowledge in pain from research to education Quintessence, Chicago. 2001;15–25.

[34] Petersson K, Finizia C, Pauli N, Tuomi L. Preventing radiation-induced dysphagia and trismus in head and neck cancer-a randomized controlled trial. Head Neck. 2024. 10.1002/hed.27886. 39091121 10.1002/hed.27886 PMC11635747

[35] Pocock SJ. Allocation of patients to treatment in clinical trials. Biometrics. 1979;183–97. 497334

[36] Bar-Ad V, Palmer J, Yang H, Cognetti D, Curry J, Luginbuhl A, et al. Current management of locally advanced head and neck cancer: the combination of chemotherapy with locoregional treatments. Semin Oncol: Elsevier; 2014. p. 798–806. 10.1053/j.seminoncol.2014.09.018 25499638

[37] Pauli N, Svensson U, Karlsson T, Finizia C. Exercise intervention for the treatment of trismus in head and neck cancer–a prospective two-year follow-up study. Acta Oncol. 2016;55(6):686–92. 26878553 10.3109/0284186X.2015.1133928

[38] Pauli N, Andréll P, Johansson M, Fagerberg-Mohlin B, Finizia C. Treating trismus: a prospective study on effect and compliance to jaw exercise therapy in head and neck cancer. Head Neck. 2015;37(12):1738–44. 24986051 10.1002/hed.23818

[39] Schiffman E, Ohrbach R, Truelove E, Look J, Anderson G, Goulet J-P, et al. Diagnostic criteria for temporomandibular disorders (DC/TMD) for clinical and research applications: recommendations of the International RDC/TMD Consortium Network and Orofacial Pain Special Interest Group. J Oral Facial Pain Headache. 2014;28(1):6. 24482784 10.11607/jop.1151 PMC4478082

[40] Galantino ML, Eden MM, Spinelli BA, Flores AM. EDGE Task Force on Head and Neck Cancer Outcomes: a systematic review of outcome measures for temporomandibular-related dysfunction. Rehabil Oncol. 2015;33(2):6–14.

[41] Elliott AM, Smith BH, Smith WC, Chambers WA. Changes in chronic pain severity over time: the Chronic Pain Grade as a valid measure. Pain. 2000;88(3):303–8. 10.1016/s0304-3959(00)00337-7. 11068118 10.1016/S0304-3959(00)00337-7

[42] Von Korff M, Ormel J, Keefe FJ, Dworkin SF. Grading the severity of chronic pain. Pain. 1992;50(2):133–49. 1408309 10.1016/0304-3959(92)90154-4

[43] Goldstein M, Maxymiw WG, Cummings BJ, Wood RE. The effects of antitumor irradiation on mandibular opening and mobility: a prospective study of 58 patients. Oral Surg Oral Med Oral Pathol Oral Radiol Endodontol. 1999;88(3):365–73. 10.1016/s1079-2104(99)70044-2 10503870

[44] Wang CJ, Huang EY, Hsu HC, Chen HC, Fang FM, Hsiung CY. The degree and time-course assessment of radiation-induced trismus occurring after radiotherapy for nasopharyngeal cancer. Laryngoscope. 2005;115(8):1458–60. 16094124 10.1097/01.mlg.0000171019.80351.46

[45] Gupta T, Agarwal J, Jain S, Phurailatpam R, Kannan S, Ghosh-Laskar S, et al. Three-dimensional conformal radiotherapy (3D-CRT) versus intensity modulated radiation therapy (IMRT) in squamous cell carcinoma of the head and neck: a randomized controlled trial. Radiother Oncol. 2012;104(3):343–8. 22853852 10.1016/j.radonc.2012.07.001

[46] Köhler AA, Hugoson A, Magnusson T. Clinical signs indicative of temporomandibular disorders in adults: time trends and associated factors. Swed Dent J. 2013;37(1):1–11. 23721032

[47] Lindfors E, Magnusson T, Ernberg M. Effect of therapeutic jaw exercises in the treatment of masticatory myofascial pain: a randomized controlled study. J Oral Facial Pain Headache. 2020;34(4). 10.11607/ofph.2670 33290442

[48] Shimada A, Ogawa T, Sammour SR, Narihara T, Kinomura S, Koide R, et al. Effectiveness of exercise therapy on pain relief and jaw mobility in patients with pain-related temporomandibular disorders: a systematic review. Front Oral Health. 2023;4. 10.3389/froh.2023.1170966 PMC10382173 37521175

[49] Idáñez-Robles AM, Obrero-Gaitán E, Lomas-Vega R, Osuna-Pérez MC, Cortés-Pérez I, Zagalaz-Anula N. Exercise therapy improves pain and mouth opening in temporomandibular disorders: a systematic review with meta-analysis. Clin Rehabil. 2023;37(4):443–61. 36263523 10.1177/02692155221133523

[50] Høgdal N, Juhl C, Aadahl M, Gluud C. Early preventive exercises versus usual care does not seem to reduce trismus in patients treated with radiotherapy for cancer in the oral cavity or oropharynx: a randomised clinical trial. Acta Oncol. 2015;54(1):80–7. 25229260 10.3109/0284186X.2014.954677

[51] Wang Y-H, Huang Y-A, Chen I-H, Hou W-H, Kang Y-N. Exercise for trismus prevention in patients with head and neck cancer: a network meta-analysis of randomized controlled trials. Healthcare: MDPI. 2022;442. 10.3390/healthcare10030442 PMC8951417 35326920

