# Supplementary material for: Jaw exercise in head and neck cancer patients for prevention of temporomandibular disorders: a randomized controlled trial
Source: J Cancer Surviv. 2024 Nov 22;20(3):1145–56. doi: 10.1007/s11764-024-01717-w (PMC13144218; doi:10.1007/s11764-024-01717-w)
Supplement: Supplementary file 1 — Supplementary file1 (DOCX 14 KB) [file 11764_2024_1717_MOESM1_ESM.docx]

**Supplementary table 1.** Per protocol analysis. The table shows the changes of MIO in intervention and control groups and the comparison of the difference of change of MIO in intervention and control groups.

|  | Intervention group  (n = 13) | | | | | Control group  (n = 19) | | | | | Comparison of intervention  and control group  (per protocol no imputation) | |
| --- | --- | --- | --- | --- | --- | --- | --- | --- | --- | --- | --- | --- |
|  | PRE | 6M | 1Y | Change Baseline  to 6M | Change Baseline  to 1Y | PRE | 6M | 1Y | Change Baseline to 6M | Change Baseline to 1Y | Difference Baseline to 6M | Difference Baseline to 1Y |
|  | Mean ± SD  Median (Min; Max) | | | | | Mean ± SD  Median (Min; Max) | | | | | *p*-value | |
| MIO | 47.7 (7.1)  46 (36; 60)  n=13 | 47.0 (6.5)  45 (39; 60) n=13 | 49.3 (6.6)  48 (40; 60)  n = 12 | -0.7 (1.9)  0 (-4; 4)  n=13 | 1.4 (5.6)  0 (-2; 19)  n = 12 | 50.8 (5.7)  50 (43; 61) n=19 | 45.2 (6.4)  45 (38; 60) n=19 | 42.3 (6.5)  45 (39; 60)  n = 18 | -5.6 (3.6)  -5 (-10;0) n=19 | -4.6 (3.4)  -5 (-10; 0)  n = 18 | < 0.001 | < 0.001 |
| MIO = Maximal Interincisal Opening | | | | | | | | | | | | |
